# A Four-Wavelength Flow-Through Fluorescence–Scatterometric Sensor That Allows for Real-Time Determination of Fat and Protein Content in Milk–Air Mixtures with High Accuracy

**DOI:** 10.3390/s26092894

**Published:** 2026-05-05

**Authors:** Maxim E. Astashev, Dmitry N. Ignatenko, Elena A. Molkova, Ivan M. Gogolev, Andrey V. Onegov, Sergey Y. Smolentsev, Artem R. Khakimov, Semen S. Ruzin, Dmitry A. Budnikov, Dmitriy Yu. Pavkin, Sergey V. Gudkov

**Affiliations:** 1 Federal Scientific Agroengineering Center VIM, 109428 Moscow, Russia; astashev@yandex.ru (M.E.A.); dmitriyek13104@yandex.ru (D.N.I.); arty.hv@gmail.com (A.R.K.); ruzin.s.s@yandex.ru (S.S.R.); dimm13@inbox.ru (D.A.B.); dimqaqa@mail.ru (D.Y.P.); 2 Prokhorov General Physics Institute of the Russian Academy of Sciences, Vavilov Str. 38, 119991 Moscow, Russia; bronkos627@gmail.com; 3 Department of Fundamental Sciences, Bauman Moscow State Technical University, 5, 2nd Baumanskaya St., 105005 Moscow, Russia; vania.gogolew@gmail.com; 4 Mari State University, pl. Lenin 1, 420000 Yoshkar-Ola, Mari El Republic, Russia; a.onegov@mail.ru (A.V.O.); smolentsev82@mail.ru (S.Y.S.)

**Keywords:** flow milk sensor, fat, protein, scatterometry, fluorometry

## Abstract

(1) Background: Currently, there is a problem of prompt determination of fat and protein content in the milk–air mixture of milking machines. (2) Methods: A design of a sensor prototype is proposed, combining measurements of light scattering (scatterometry) and fluorescence (fluorometry) to determine the component composition of the milk–air mixture formed during milking. (3) Results: An optical and electronic circuit of a flow sensor has been developed, using four sources of optical radiation: blue, green and red semiconductor lasers (light scattering in milk) and a UV LED (milk fluorescence), as well as an axial photodiode array for recording the light scattering indicatrix and the fluorescence intensity of the milk–air mixture. The use of three laser sources in the scatterometric circuit allows for the determination of the fat content in milk with an error of 0.05%, which is better than all currently known analogs. The developed sensor enables the detection of counterfeit milk containing palm oil instead of milk fat. It operates reliably in a temperature range of 5–35 °C and at milk flow rates of up to 100 mL/sec. (4) Conclusions: The sensor is capable of transmitting real-time data on the fat and protein content of milk to an RS-232 serial port, enabling integration into milking robots and automated milking systems.

## 1. Introduction

Milk is a biological fluid, a product of normal physiological secretion of the mammary glands of female mammals, produced during lactation [1]. Evolutionarily and physiologically, milk is intended for breastfeeding and nutrition [2]. Milk is an aqueous (86–88% water) colloid containing fats (3–6%), proteins (2–4% with the highest percentage of casein), lactose (3–6%) and mineral salts [3,4,5]. From the point of view of the interaction of milk components with optical radiation, milk is a multicomponent dispersed highly scattering medium [6]. The market value of milk depends mainly on the fat and protein content [7]. Rapid analysis [8,9] of the percentage content of these milk components, as well as lactose, amino acids, progesterone, and microbiological impurities, provides the necessary information to assess milk quality indicators, which in turn makes it possible to adjust the feed balance for cows and diagnose their clinical condition [10].

Rapid quantitative assessment of milk composition can be performed using various methods [11]. In particular, milk analyzers based on Fourier transform infrared spectroscopy (FTIR) exist; these analyzers are very expensive and typically bulky [12]. In addition to FTIR systems, less expensive and more compact near-infrared (NIR) spectroscopic milk analyzers are increasingly being used [13]. These methods allow one to determine the concentration of both fat and protein in milk. While their accuracy is quite high, they do not allow for flow measurements, and measuring a single sample takes a long time (tens of seconds) and requires expensive equipment (e.g., 10,000 USD). A separate category of methods allows one to measure only the protein concentration in milk—the Bretford, Kjeldahl, Lowry, and other methods. These methods require complex sample preparation and a long measurement time (tens of minutes) for a single sample. Compared with spectroscopic instruments, devices based on light scattering (scatterometric devices) offer a number of important advantages. Such devices can be made smaller and more compact. They do not contain expensive components, meaning they can be manufactured inexpensively. Measuring scatterometric parameters is a relatively fast process, meaning such devices can obtain information in real time. At the same time, scatterometric measurement technology is scalable in complexity, and there are also sophisticated scatterometric solutions, such as photon density wave spectroscopy (PDW) [14], which allow obtaining additional information, for example, particle size distribution in highly scattering media. Our proposed approach is similar to static multiple light scattering (SMLS) [15], but is specifically tailored for working with milk flow in milking systems and for determining the concentration of scattering particles. Previously, our team developed a milk fat analyzer based on single scattering; such an analyzer could not work with whole milk, and it was necessary to dilute the milk with deionized water before measurement [16]. To date, several devices have been proposed that use scatterometry to determine the fat content of whole milk [17,18,19], as well as the detection of somatic cells as an indicator of mastitis, even under difficult conditions such as milk–air mixtures [20]. Unfortunately, the margin of error of currently known solutions for determining the milk fat content in a milk–air mixture passing through milking systems is at least 0.2%, which is clearly insufficient. The objective of developing the proposed device was to ensure its integration at the milking machine level and to keep the cost of the solution at the level of 300 USD, which was achieved. This study proposes a new approach to diagnosing the particle composition of milk, based on the analysis of light scattering from three independent radiation sources of different wavelengths. This approach, despite slightly increasing the analysis time to 50 s per measurement period (10 s each for the three lasers, UV diode, and background), significantly increased the information yield of the measurement, allowing for the detection of not only milk fat concentration but also the content of other scattering particles. While maintaining the ability to analyze the profile of these parameters’ changes during milking, allowing for approximately 20 individual measurements. The sensor integrates a developed solution that detects the fluorescent signal from protein molecules, which also enables the measurement of milk protein concentration.

## 2. Materials and Methods

### 2.1. Sensor

**Figure 1:**
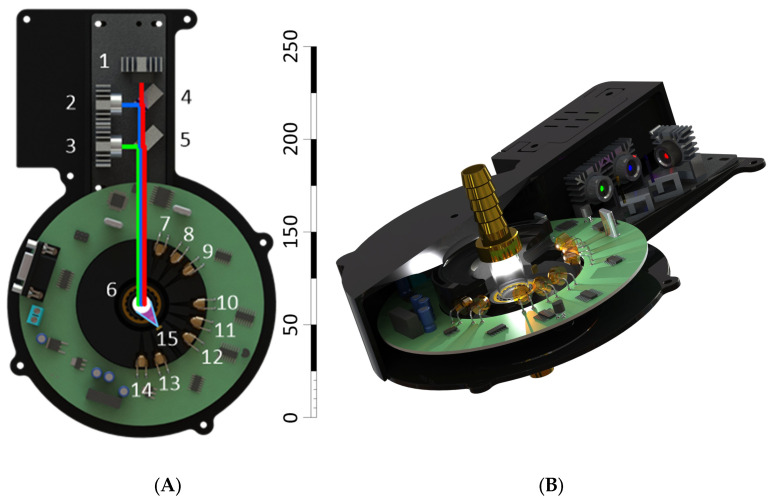
Flow sensor for determining the fat and protein content in milk. (**A**) Schematic diagram of the experimental setup for the laser scatterometer with three radiation wavelengths. Scale is shown in millimeters. The numbers indicate the following: 1—red laser with a wavelength of 625 nm and a power of 5 mW. 2—green laser with wavelength of 532 nm and power of 5 mW. 3—blue laser with wavelength of 450 nm and power of 5 mW. 4—dichroic mirror with a separation wavelength of 580 nm. 5—band dichroic mirror with a reflection wavelength of 540 nm (band width 25 nm). 6—cylindrical cuvette with an outer diameter of 15 mm, wall thickness of 1 mm, made of fused quartz. 7–14—silicon photodiodes FD263-01. 15—UV LED with an emission wavelength of 280 nm. (**B**) External appearance of the flow sensor for determining the fat and protein content in milk.

The experimental setup of a laser scatterometer with three wavelengths of radiation is shown in Figure 1. The device is a laboratory prototype for refining the optical and electronic recording circuitry. It offers ample room for future upgrades, including the ability to use digital filters to address the issue of measurement interruptions when passing air pockets. Serious oversampling is provided for this purpose. The primary requirement for lasers is output power stability, as the measurement algorithm sequentially polls photodiodes when changing wavelengths and then compares levels. We use a standard LRS-350-12 power supply (Mean Well Enterprises Co., Ltd., New Taipei City, Taiwan); the device’s consumption does not exceed 1 A DC. The device’s operating time is limited only by internal contamination of the cuvette. Milk tends to accumulate components on the walls of milk pipelines and other fittings. However, in food production, periodic flushing is required using appropriate Clean-In-Place systems. Radiation from three semiconductor laser diodes of blue, green and red colors (numbers 1–3 in Figure 1A) is combined into one optical path using dichroic mirrors (numbers 4–5 in Figure 1A) and irradiates a cuvette (number 6 in Figure 1A) with the suspension under study in sequential mode. The cuvette is a transparent cylindrical quartz tube (outer diameter of 15 mm and wall thickness of 1 mm). The ends of the tube are terminated with fittings for connecting milk hoses. The angular distribution of the scattered light intensity is recorded using eight photodiodes (numbers 7–14 in Figure 1A), arranged circumferentially at 18° angular increments. The positions between LEDs 9 and 10, as well as 12 and 13, are left empty for the installation of additional light sources, but this does not affect the evaluation of the measured parameters. As a result, three groups of photodiodes are formed: forward (0° and 18°), side scattering (54°, 72°, and 90°), and back scattering (126°, 144°, and 162°). Before reaching the photodiodes, the scattered light field is limited by 1 mm wide rectangular diaphragms made in a plastic ring. The photodiodes operate in photoconductive mode. When measuring the protein content, an ultraviolet LED with an emission wavelength of 280 nm (number 15 in Figure 1A) is turned on, irradiating the measuring cuvette. The luminescence intensity is recorded by the closest photodiode (number 12 in Figure 1A). The LED photocurrent is amplified using a transimpedance amplifier based on the OP482 op amp (Texas Instruments Incorporated, Dallas, TX, USA). After amplification, the photodiode signals are converted to digital form using an E14-140 ADC (L-Card, Moscow, Russia) module, which has a single analog-to-digital converter paired with an eight-channel multiplexer. The analog-to-digital converter’s sampling frequency is 100 kHz. After the multiplexer, each of the eight channels is digitized at a frequency of 100/8 = 12.5 kHz. Ten seconds of sample exposure to each laser produces 125,000 digitized measurements, which are filtered and averaged. The 10 s laser illumination period is chosen so that 3–5 milking periods occur in a standard automatic milking system, and the same number of milk filling and air pocket clearance periods occur in the milk pipeline. This should allow for the subsequent elimination of air pockets using a digital filtration system. Figure 1B shows a cross-sectional view of the sensor.

### 2.2. Operating Algorithm

**Figure 2:**
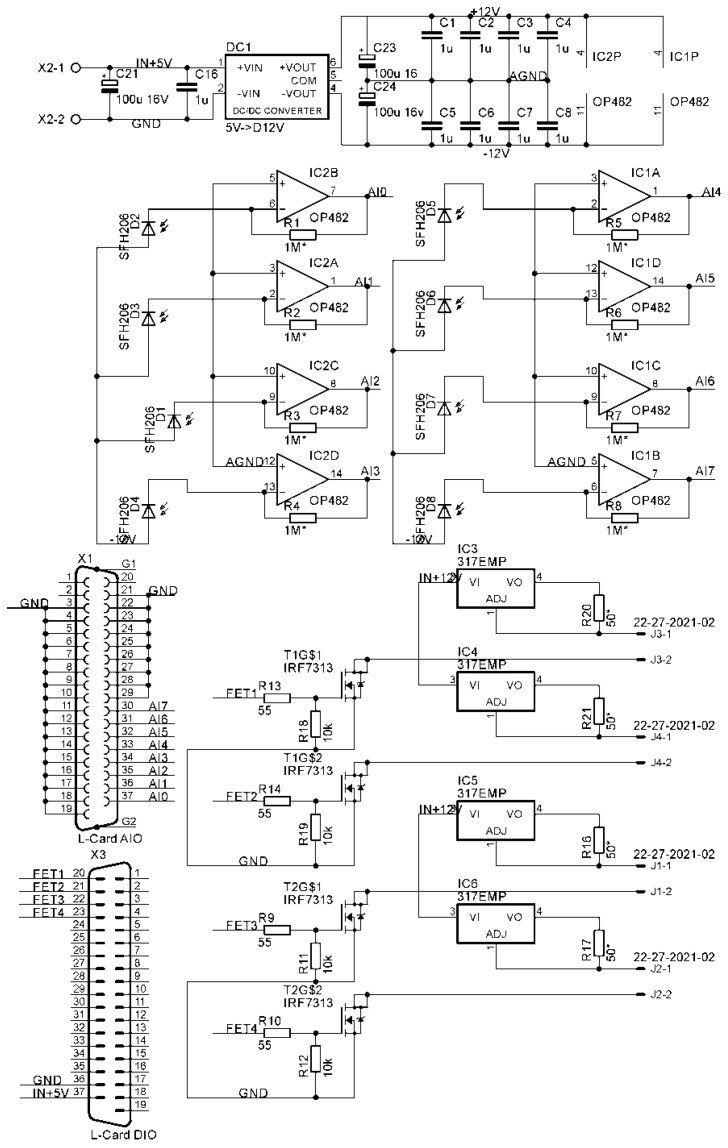
Electronic circuit diagram of the flow sensor for determining the fat and protein content in milk. IC1 and IC2 are operational amplifiers of the current-to-voltage converters (transimpedance amplifiers). IC3-IC6 are current stabilizers of the laser diodes and UV LED. T1 and T2 are dual FET transistors for controlling light sources. D1-D8 are photodiodes. X1 and X3 are connectors for connecting analog (X1) and digital (X3) lines to the L-Card E14-440 ADC. DC1 is a DC-DC converter that converts 5 V to a bipolar voltage of ±12 V for powering the analog amplifiers. The In +12 V line is connected to an external 12 V, 2 A power supply. * The asterisk indicates that the indicated resistance values are approximate and can be replaced based on the results of testing and calibration in case of overload of the transimpedance amplifier.

Figure 2 shows the electronic circuit diagram of a flow-through sensor for determining fat and protein content in milk. It consists of a bipolar power supply system based on DC-DC converter DC1, transimpedance photocurrent amplifiers based on phased amplifiers IC1 and IC2, light source current regulators based on IC3-IC6 microcircuits, and FET light source control drivers T1 and T2. Connectors X1 and X3 are used to connect to the analog and digital lines of the L-Card L-14-440 (OOO L-card, Moscow, Russia) acquisition unit. The laser diode illumination control signals are generated by the digital outputs of the E14-140 TTL ADC. The measurement sequence in fat content determination mode is as follows: (1) no light, (2) only the red laser diode is illuminated, (3) only the green laser diode is illuminated, (4) only the blue laser diode is illuminated. The ignition time of each diode is 10 s. The entire measurement period is 40 s. During the illumination time of each LED, approximately 125,000 measurements are obtained. The sample time series is first filtered with a median filter with a window length of 16 to exclude outliers. Then, the data were averaged to increase measurement accuracy. The measurement sequence in protein content determination mode is as follows: (1) no light, (2) the ultraviolet LED is illuminated. The diode ignition time is 10 s. The entire measurement period is 20 s. During the LED’s illumination time, approximately 8000 measurements are taken, which are first filtered with a median filter with a window length of 4 and then averaged to improve measurement accuracy. It was decided to reduce the sampling frequency, since the fluorescence measurement actually occurs in a fairly thin film, which remains on the cuvette stacks throughout the entire measurement time, and air bubbles and foaming do not interfere as much as when recording scattering. The high sampling rate for scatterometer data obtaining will allow future data filtering to address artifacts caused by air pockets in the milk line. Obtaining “dark” data allows for compensation for external light pollution by subtracting the “dark” values from the values obtained during illumination of the corresponding laser diode. The resulting scattered light data is then used to estimate milk fat content using a personal computer program.

### 2.3. Milk Sources

Commercially available, fat-standardized, ultra-pasteurized, homogenized milk samples from different manufacturers with 1% and 6% fat content were used as samples for comparing the performance of the proposed measuring device. Five samples from different batches of Tetrapak milk were used. Intermediate concentrations were obtained by mixing these samples. Cream with a 10% fat content was also purchased in ultra-pasteurized form. Fresh milk of the Kholmogory breed was also used in some experiments.

Mixtures of pure milk fat, casein protein, lactose, and saline solution were used as reference samples. Milk fat samples were isolated from whole milk by centrifugation at 3500 rpm with a rotor radius of 12.5 cm and a run time of 30 min. α-Casein from cow’s milk (Sigma-Aldrich, St. Louis, MI, USA) and lactose monohydrate (CDH, New Delhi, India) were also used as reference samples in the experiments. The initial concentrations of fat, protein, and lactose in the calibration colloids and solutions were determined by weighing. Fat and proteins were sonicated. Micelle size was monitored using Malvern Red Label dynamic light scattering (Malvern Panalytical, Malvern, UK).

### 2.4. Auxiliary Equipment

To simulate milk flow in real milk pipelines, a 2 L thermostatic tank (CYY-2, Shenzhen, China) was used, connected to an electric pump. Milk consumption was determined using the volumetric method based on the time it took to fill a 1 L measuring cylinder. A hose from the electric pump was inserted into the inlet of the developed sensor. After passing the sensor, milk returned to the thermostatic tank. Milk flowed through the system at velocities ranging from 0 to 120 mL/s. An 8300 fluorimeter (Jasco, Tokyo, Japan) with a shutter was used to create 3D fluorescence maps. Approximately 3 mL of milk was analyzed at a time. Milk fat content and protein content were determined using a Lactan 600 Ultra (extended) milk quality analyzer (OOO VPK Sibagro pribor, Novosibirsk, Russia). Modifications were made to the transducer and receiver. The device’s reproducibility for measuring both fat and protein is approximately 0.01%. The device’s accuracy was monitored using standard solutions. A Sibir 2-PL electric separator (Omsk Separator Plant, Omsk, Russia) was used for milk separation.

### 2.5. Statistical Data Analysis and Visualization

The results on the graphs are presented as mean values. In some cases, in the form of mean and standard errors of the mean. The number of independent repetitions is at least five. By independent repetition, we mean the result of an experiment on samples manufactured de novo.

## 3. Results

### 3.1. Measuring Fat Content in Milk

Light scattering characteristics in a milk cuvette were determined using the scattered light intensity function measured at different angles, i.e., the scattering indicatrix. The angle was determined between the laser beam direction and the line from the center of the cuvette to the detector window. The scattering indicatrices were measured for commercially available cow’s milk samples with fat contents of 1.0%, 1.5%, 3.5%, 6.0%, and 10%, respectively. Three probe laser diodes with wavelengths of 450, 532, and 625 nm were used. The laser beam passed through the center of the milk cuvette. An axial array of photodiodes surrounded the cuvette, positioned at angles to the laser beam axis (0°, 18°, 54°, 72°, 90°, 126°, 144°, and 162°). Milk of varying fat content was placed in the cuvette without milk flow. It was found that the scattered radiation intensity decreases with decreasing scattering angle. Given that weak scattering theory is not valid in this case, as milk is a highly scattering medium for radiation propagation, this dependence is more accurately described as follows: the radiation intensity decreases with increasing distance from the radiation entry point into the sample. Moreover, this dependence is linear for the logarithm of the photocurrent.

**Figure 3:**
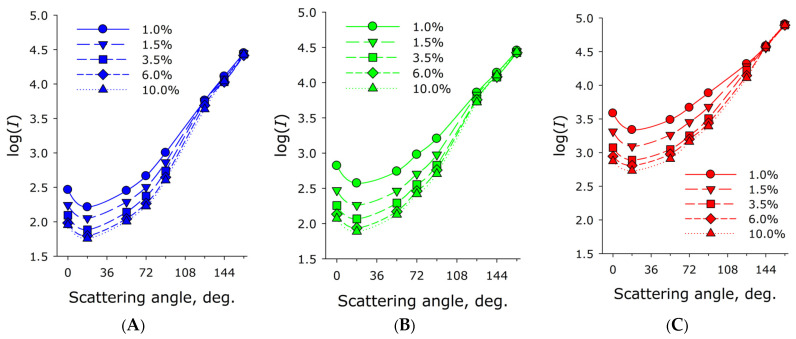
Scattering indicatrices measured for milk samples with nominal fat contents of 1.0%, 1.5%, 3.5%, 6%, and 10% at 20 °C. The milk has zero flow velocity (stationary). The indicatrices are presented on a logarithmic scale. The wavelength of the probing laser light is 450 nm (**A**), 532 nm (**B**), and 625 nm (**C**). Data are presented as mean values ± standard error of the mean. N = 5. The standard error of the mean for the logarithm of the scattering intensity is less than 0.02 (not visible behind the dots). Each point on the graphs is based on three independent repetitions.

Figure 3 shows the scattered radiation indicatrix diagrams for the logarithms of the scattered radiation for milk samples containing different fat concentrations. It was found that the scattered radiation intensity when probing the samples with a red laser is significantly higher than when probing with blue or green light. Also noteworthy is the lower selectivity (the range of measurements between milk samples with different fat contents) when irradiating the sample with a blue laser.

The slope of the scattering indicatrix was approximated using the least-squares method, yielding the following relationship:

(1) $$Log\left(I_{i}\left(\alpha \right)\right)={\alpha x}_{i}+b$$

where *I_i_*(*α*) is the photocurrent of a photodiode positioned at angle *α* when illuminated by light source number *i* (*i* = 1–3 for red, green, and blue lasers, respectively); *x_i_* is the slope of the scattering indicatrix, which characterizes the fat content of the milk; and *b* is the offset, which can be eliminated by appropriate normalization of the photodiode signals.

**Figure 4:**
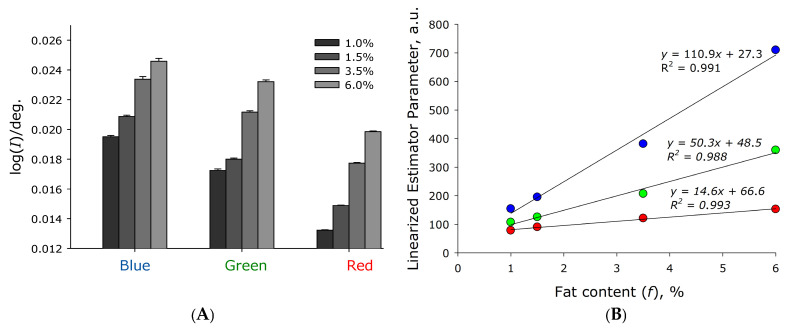
Chart of the logarithmic scattering function in milk with different fat contents when probed with laser sources with wavelengths of 450, 532, and 625 nm. (**A**) Dependence of the slope of the logarithmic scattering function (in the angular range of 72–162°) on the fat content of milk. (**B**) Linearization of the slope of the logarithmic scattering function on the fat content of milk. Data are presented as mean values ± standard error of the mean. N = 5.

Figure 4A shows the slope of the logarithmic scattering indicatrix in milk with different fat contents when probed with laser sources with wavelengths of 450, 532, and 625 nm. It was found that, when probing milk with different lasers, instability in the obtained results increases in the order red laser, green laser, and blue laser. The standard errors of the mean for red laser probing are at least four times smaller than for blue laser probing. Overall, the slope of the scatter plot increases approximately sixfold as fat content increases from 0 to 6%.

The dependence of the scattering indicatrix slope on milk fat content can be linearized. In this study, the simplest linearization approach was used. The hyperbolic function was

*y_i_* = 1/(*A* − *x_i_*) (2)

where *x_i_* is the slope of the scattering indicatrix for the *i*-th radiation source, *i* = 1–3 for the red, green, and blue lasers, respectively; *y_i_* is the linearized estimator parameter for the *i*-th radiation source. It was found that, when probing with all studied laser diode types, a linearization of the parameters obtained on the experimental setup versus milk fat content was observed (Figure 4B). Moreover, the hyperbolic parameter had the same value, A = 0.026, for all tested laser diode wavelengths, indicating its dependence on the experimental setup geometry, cuvette diameter, and photodiode sensitivity, but not on the scattering conditions in the sample. The dependence of the parameter *y_i_* on the fat content of milk can be expressed as a linear relationship:

(3) $$y_{i}={fB}_{i}+C_{i}$$

where *f* is the fat content of milk in percent, and *B_i_* and *C_i_* are wavelength-dependent linearization parameters. The dependence of the parameters on the wavelength of the probing radiation is shown in Table 1.

The fat-independent regression offset parameter *C_i_* is the scattering parameter in milk at zero fat content, meaning it is determined by scattering and absorption in proteins, cells, and other particles, excluding fat in the milk suspension. It can be seen that this parameter decreases with decreasing wavelength, indicating a decrease in scattering and, therefore, a shift away from maximum scattering, i.e., the size of non-fat scattering particles is larger than the wavelength of the probing radiation.

Overall, using a single laser module, the error of determining the fat concentration in the milk–air mixture is less than 0.2%. Using all three laser modules, the reproducibility of determining the fat concentration is approximately 0.05%, which is better than all currently known analogs. Figure 4B does not show values for 10% fat, as at this fat content, a significant deviation from linearization is observed (the values are smaller than expected). Overall, the function can be linearized with the required accuracy only for the red laser. The observed lower values for samples with a fat concentration of 10% indicate that the decrease in the relative number of scattering particles in the high-fat product may be due to the larger size of the fat particles. However, a fat concentration of 10% is significantly higher than the fat range of unprocessed cow’s milk and was used as a test of the upper limit of fat content measurement by the sensor.

**Figure 5:**
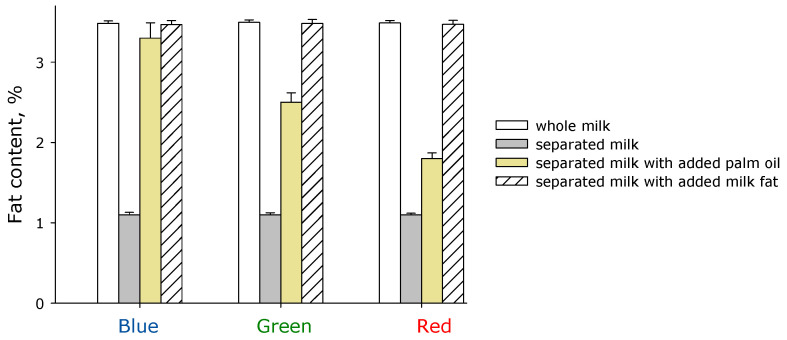
Measuring the fat concentration in milk using the developed sensor. The results obtained by probing the samples with blue, green and red laser beams are presented. Measured samples: 1. Whole milk; 2. Separated milk; 3. Separated milk with added palm oil to a final fat concentration of 3.5%; 4. Separated milk with added milk fat to a final concentration of 3.5%. Data are presented as mean values ± standard error of the mean. N = 5.

The proposed method for assessing milk fat content based on the optical properties of the scattering medium enables the detection of particles of various sizes in suspension, making it possible to detect the addition of foreign components, vegetable fats, and cellular contamination to milk. A series of experiments was conducted in which the fat concentration in milk was measured using the developed sensor. The results obtained by probing the samples with blue, green, and red laser radiation are presented (Figure 5).

It is shown that whole milk with a nominal fat content of 3.5% is defined as milk with a fat content of 3.5% when probing at all wavelengths. After passing whole milk through a separator equipped with 9 of 12 separation plates, approximately 1% fat remains in the milk, which is also confirmed by probing at all wavelengths. When adding palm oil to skimmed milk containing approximately 1% fat to achieve a final fat concentration of 3.5%, significant deviations from the nominal value were observed. It should be noted that palm oil was dispersed in the milk using ultrasound while heating slowly, after which the resulting milk product was left to settle for an hour. When determining the fat content of milk with added palm oil using a blue laser, the sensor indicated a fat content of 3.3%, which is generally close to the nominal value. However, when determining the fat content using a green laser, the sensor determined a fat content of 2.5% in milk with added palm oil. When using a red laser, the sensor indicated a fat content of less than 2%, demonstrating a monotonic dependence of the fat determination error. It cannot be ruled out that the obtained results are due to a suboptimal fat transfer system into the milk. Therefore, a series of experiments was conducted in which separated milk fat was added to milk that had passed through a separator to achieve a final concentration of 3.5%. It was found that in milk that has passed through a separator with subsequent addition of milk fat to a final concentration of 3.5%, the sensor detects 3.47–3.48%.

**Figure 6:**
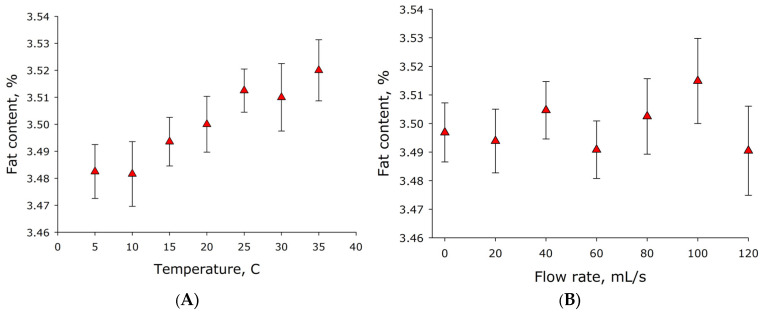
Sensor readings as a function of milk temperature (**A**) and milk flow through the measuring cuvette at different speeds (**B**). Probing with a 625 nm laser source. Nominal milk fat content is 3.5%. Data are presented as mean values ± standard error of the mean. N = 5.

Under real-life dairy farm conditions, milk temperature in hoses and pipelines can vary significantly at different times of year or with different room process parameters. The effect of temperature on milk fat content sensor readings was studied over a temperature range of 5–35 °C (Figure 6A). Measurements were conducted using milk with a fat content of 3.5%. It was shown that milk fat sensor readings tend to increase with increasing temperature. Moreover, sensor readings vary by just over 1% over temperature changes from 5 to 35 °C. The average change in sensor readings is approximately 0.0013%/°C. It can be assumed that temperature has an insignificant effect on the measured milk fat content.

### 3.2. Measuring Milk Protein Content

Under real-world conditions, milk in hoses and pipelines is constantly moving. The effect of milk flow rate on milk fat sensor readings was studied (Figure 6B). Milk flow rate through the sensor varied in the range of 0–120 mL/s. Sensor readings were measured for 2 min at each flow rate. Five cycles of all flow rates within the range were conducted. It was shown that changing milk flow rate in the range of 0–120 mL/s does not significantly affect the measured parameter. However, with increasing flow rate, a tendency for the standard errors of the mean to increase was observed.

**Figure 7:**
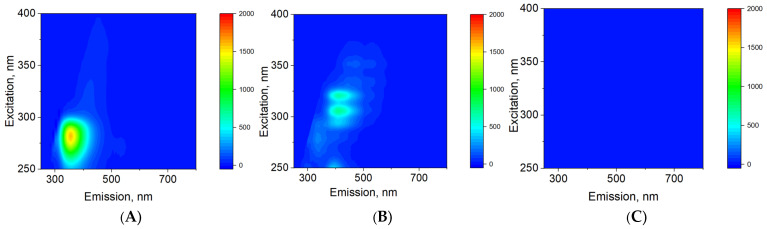
3D fluorescence maps of α-casein (**A**), milk fat (**B**) and lactose (**C**).

The fluorescence of milk fat, α-casein, and lactose—the main components of milk—was studied (Figure 7). Only casein and milk fat were found to exhibit intense fluorescence. Lactose did not exhibit fluorescence. The milk fat fluorescence region corresponded to excitations with wavelengths of 300–330 nm, with emission observed at wavelengths of 370–490 nm. The casein fluorescence region corresponded to excitations with wavelengths of 270–285 nm, with emission observed at wavelengths of 305–400 nm. It was shown that the fluorescence maxima of milk fats and milk proteins do not overlap in the spectral range. The overlap in the total intensities of the fluorescence regions is no more than 0.03. Thus, direct measurement of milk proteins is possible using fluorescence signal scattering.

**Figure 8:**
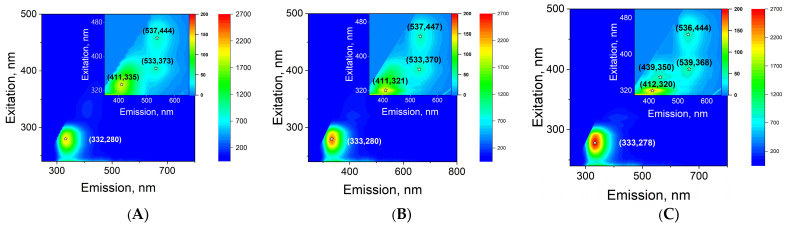
3D fluorescence maps of milk containing 1.0% fat and 3.17% protein (**A**), 3.5% fat and 3.61% protein (**B**), and 6.0% fat and 4.43% protein (**C**). ✩—maximum fluorescence intensity, numbers indicate emission and excitation wavelengths.

The fluorescence of several milk samples with known fat and protein contents was studied (Figure 8). Casein is clearly visible in the milk samples. All maps show a fluorescence scattering region with coordinates λ_ex_ 270–285 nm and λ_em_ 305–400 nm. With increasing protein concentration, the casein fluorescence intensity increases. The fluorescence region characteristic of fat molecules is difficult to discern in all fluorescence maps, although it is present. Several “minor” fluorescence maxima are also detected under long-wavelength excitation. These fluorescence maxima have coordinates λ_em_/λ_ex_ = 411/330 nm, λ_em_/λ_ex_ = 533/370 nm, and λ_em_/λ_ex_ = 533/445 nm. The intensity of these fluorescence scattering maxima is approximately one order of magnitude lower than the fluorescence intensity of milk protein.

**Figure 9:**
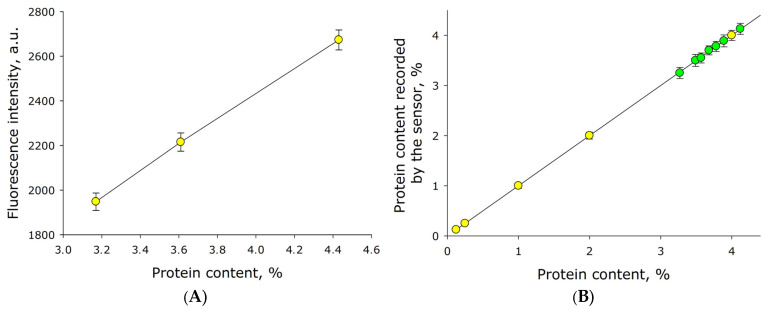
Dependence of the maximum fluorescence intensity of casein on its concentration in milk. (**A**) Intensity of the maximum fluorescence of milk with different casein concentrations from Figure 8, measured by the developed sensor. (**B**) Intensity of the maximum fluorescence of milk, depending on the degree of its dilution with water. Yellow dots represent different protein concentrations obtained by diluting the same milk samples with water. Green dots represent different milk samples from the same batch. Measured values obtained by the developed sensor. The original milk is taken as 100%. N = 5.

The effect of milk protein concentration on the fluorescence signal intensity with coordinates λ_ex_ 280 nm and λ_em_ 333 nm was studied. The same milk samples as in Figure 8 (Figure 9A) were measured. It was shown that the fluorescence intensity recorded by the sensor is linearly dependent on the milk protein concentration. The resulting data points are linearized by the following equation:

*y* = 273*x* + 136 (4)

where *y* is the normalized fluorescence intensity (in arbitrary units), and *x* is the protein concentration (in mass %). Comparative tests were conducted in which milk with a known protein concentration was diluted with water by 2, 4, 16, and 32 times (Figure 9, yellow dots). It was shown that the milk protein concentration detected by the sensor decreases linearly with each dilution. In general, the function can be described by the linear equation:

*y* = *x* (5)

where *x* is the milk protein concentration (in mass %) measured by the reference instrument, and *y* is the protein concentration (in mass %) measured by the developed sensor. Also shown in Figure 9 are the points representing the measured protein concentration in raw milk, shown in green. Overall, the error of milk protein concentration determination is no more than 0.12%.

**Figure 10:**
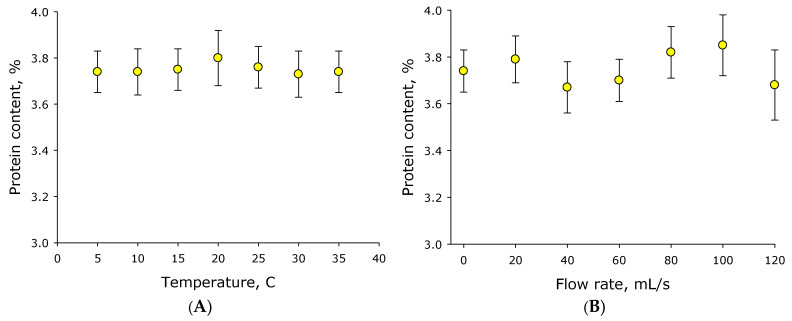
The influence of external conditions on the protein concentration measured by the sensor. Sensor readings as a function of milk temperature (**A**) and milk flow rate through the measuring cuvette (**B**).

The effect of temperature on the protein concentration measured by the sensor was studied (Figure 10A). Measurements were conducted on milk with a casein content of 3.74%. It was shown that temperatures in the range of 5–35 °C do not affect the casein content measured by the sensor, nor does it affect the accuracy of this measurement.

Milk in hoses and pipelines is typically in motion, not stationary. The effect of milk flow rate on the protein content determined by the sensor was studied (Figure 10B). Milk flow rate through the sensor was varied in the range of 0–120 mL/s. Sensor readings were recorded for 1 min at each milk flow rate. Three cycles of all flow rates in the studied range were conducted. It was shown that changing milk flow rate in the range of 0–120 mL/s does not significantly affect the determined casein content in the milk. At the same time, with increasing speed, there is a tendency for the standard errors of the mean to increase; in other words, with increasing speed, the number of incorrect detections increases.

## 4. Discussion

Optical (Figure 1) and electronic (Figure 2) circuits of the fluorescence–scatterometric four-wave flow sensor have been developed, which allow for a comprehensive analysis of milk in real time: to determine with high accuracy the content of fat and protein in the milk–air mixture, while differences in the results of assessing the parameters of light scattering at different wavelengths indicate the possibility of assessing the size of scattering particles. It has been established that the scattered radiation intensity when probing samples with red laser is significantly higher than when probing with green light, and higher for green than for blue (Figure 3). This is presumably due to the higher sensitivity of the FD263-01 silicon photodiode (Figure 2) in the red spectral region. Furthermore, lower selectivity has been demonstrated when irradiating the sample with blue and green lasers, compared to a red laser, as well as a wider range of measurements between milk samples with different fat contents (Figure 4). Importantly, in the linearization model, parameter *A* remains constant for all probing wavelengths (Table 1). This means that this parameter does not depend on the scattering conditions in the milk sample, while the parameter can be affected by the geometry of the experimental setup and cuvette, as well as possibly the sensitivity of the photodiodes.

It is known from the literature that milk contains fat micelles with sizes ranging from 0.1 to 15 μm [21]. In this case, two micelle fractions dominate in the size distribution: relatively small with characteristic sizes of 0.1–0.2 μm and relatively large with characteristic sizes of 2–4 μm [22]. It should be noted that the number of relatively large fat micelles in milk is 2–4 orders of magnitude smaller than that of small micelles [22]. The main contribution to the scattering of visible light by milk is made by relatively small micelles; however, the contribution to the overall scattering intensity of relatively large fat micelles is noticeable [23]. Casein is a collective name for several milk phosphoproteins with similar structures [24]. In the presence of hydrated calcium ions, casein molecules form primary casein spherical micelles with a size of approximately 0.01 μm [25]. Such casein micelles often aggregate into larger secondary micelles [26]. These micelles can have various shapes, but the packing in such micelles is quite dense, and the average size is approximately 0.1 μm [27]. These figures indicate a possible better correspondence between the wavelength of the red (long-wave) source and at least the size of fat particles in milk.

It is known that with increasing fat content in milk, the size of the relatively large fraction of fat micelles can increase [28]. In our experiments, the slope of the logarithm of the scattering indicatrix is most sensitive when estimating the slope at small scattering angles (Figure 3). This indicates a deviation of the scattering model in the case of significantly different fat particle sizes. An important consequence of this fact is the possibility of using the sensor we developed to detect and determine the concentration of counterfeit impurities, such as palm oil (Figure 5). It is known from the literature that optical circuits based on recording the intensity of scattered radiation in milk can detect even larger impurities, such as somatic cells (approximately 20 µm in size) in mastitis [29].

It was found that the fat content of milk, recorded by the sensor, tends to increase with increasing temperature from 5 to 35 °C (Figure 6A). This process is clearly unrelated to the operation of the electronics, as electronic components do not change their temperature. It is also clearly unrelated to the actual fat content of the milk, as the same milk was used at all temperatures in a single experiment. It is likely that changes in temperature lead to changes in the fat micelles and their size distribution [30]. Previously, it was suggested in the literature that the size of lipid micelles should decrease with increasing temperature; similar data exist for casein micelles [31]. The phenomenon of decreasing aggregate size is likely based on an increase in the critical micelle concentration with increasing temperature [32]. This also leads to another conclusion: that the size distribution of fats does not change with milk flow rate (Figure 6B).

The fluorescence of the main components of milk was studied. It was found that α-casein fluorescence does not overlap with the fluorescence of milk fat (Figure 7). In addition, the quantum yield of casein molecules upon excitation is significantly higher than the quantum yield of milk fat components (Figure 8). The characteristic range of casein fluorescence emission is ~305–400 nm, which is due to the presence of aromatic amino acids [33]. For the developed sensor, this means that the fluorescence intensity in this spectral range can be measured by axial photodiodes used to record light scattering from fat micelles. Importantly, the fluorescence intensity is linearly dependent on the concentration of casein in milk (Figure 9). Moreover, the fluorescence intensity function is linearly dependent on the concentration of casein and intersects the ordinate axis just above zero (Figure 9A). When milk is diluted with water, a similar function passes through the coordinates (0;0) (Figure 9B). These two cases differ only in the concentration of fat in the milk. This means that the fat content in milk has, albeit insignificantly, an effect on the determination of protein concentration.

Tryptophan has the most significant fluorescence intensity compared to other aromatic amino acids (tyrosine and phenylalanine) [34]. With changing temperature, the tryptophan fluorescence maximum shifts somewhat toward the long-wavelength region [35,36]. The quantum yield of tryptophan in the temperature range of 5–35 °C changes insignificantly [37]. Moreover, the fluorescence intensity of tryptophan in the protein molecule can both increase and decrease with increasing temperature [38,39,40]. Casein, apparently, does not significantly change fluorescence intensity with changing temperature in the range of 5–35 °C (Figure 10A). The milk flow rate through the sensor in the range of 0–120 mL/s also has little effect on the protein content determined by the sensor (Figure 10B).

The design challenge of the milking machine is the alternation of four main modes in the milk tract: a solid milk section, virtually free of air, a milk–air mixture, an air lock, and then a milk–air mixture again. The frequency of these modes is approximately once every 2 s. This alternation of the sample modes in the optical cuvette allows measurements to be taken only while the milk line is completely filled with milk. We believe the high data acquisition rate will enable us to filter out these issues and conduct accurate measurements using digital signal processing. In the presented device, the sampling rate is set at 12.5 kHz, which allows for a clear description of the dynamics of scattering parameters and allows us to select the optimal correction strategy for measurements with an air–milk mixture.

## 5. Conclusions

**Figure 11:**
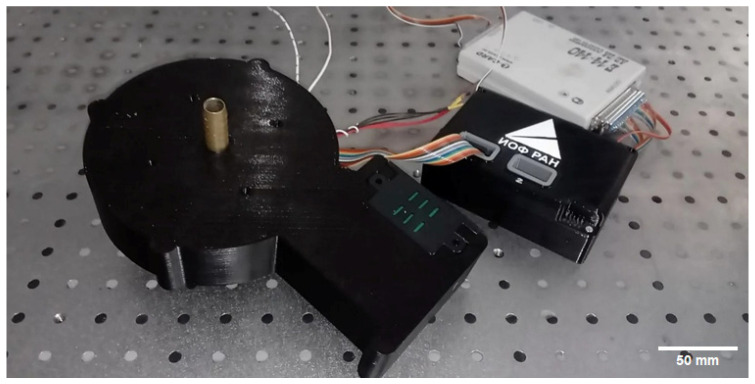
Photograph of the working sensor prototype with connected transimpedance amplifier (black block) and an analog-to-digital converter (white block).

A sensor (Figure 11) has been developed for the rapid monitoring of fat and protein content in milk. It combines light scattering (scatterometry) and fluorescence (fluorometry) measurements. The sensor is a relatively inexpensive, compact, digital device that determines the fat and protein content of milk in real time. The sensor is capable of transmitting real-time data on fat and protein content to an RS-232 serial port, enabling integration into milking robots and automated milking systems.

## Figures and Tables

**Table 1:** Dependence of linearization parameters on the wavelength of probing radiation.

Parameters	Blue	Green	Red
*A*	0.026	0.026	0.026
*B*	110.9	50.3	14.6
*C*	27.3	48.5	66.6

## Data Availability

Dataset available on request from the authors.

## References

[1] Al Sulaiti B., Ferguson-Smith A.C., Hanin G. (2025). From Mammary Glands to Nutrients: Genetic Insights into Milk Composition. Biol. Reprod..

[2] Marks M.E., Coddington Brown E.J. (2025). Mammalian Lactation as a Framework for Teaching Development, Physiology, and Cell Biology for Social Change. Dev. Biol..

[3] Plata-Pérez G., Angeles-Hernandez J.C., Morales-Almaráz E., Del Razo-Rodríguez O.E., López-González F., Peláez-Acero A., Campos-Montiel R.G., Vargas-Bello-Pérez E., Vieyra-Alberto R. (2022). Oilseed Supplementation Improves Milk Composition and Fatty Acid Profile of Cow Milk: A Meta-Analysis and Meta-Regression. Animals.

[4] Muñoz-Salinas F., Andrade-Montemayor H.M., De la Torre-Carbot K., Duarte-Vázquez M.Á., Silva-Jarquin J.C. (2022). Comparative Analysis of the Protein Composition of Goat Milk from French Alpine, Nubian, and Creole Breeds and Holstein Friesian Cow Milk: Implications for Early Infant Nutrition. Animals.

[5] Bakry I.A., Yang L., Farag M.A., Korma S.A., Khalifa I., Cacciotti I., Ziedan N.I., Jin J., Jin Q., Wei W. (2021). A Comprehensive Review of the Composition, Nutritional Value, and Functional Properties of Camel Milk Fat. Foods.

[6] Smirnova A., Konoplev G., Mukhin N., Stepanova O., Steinmann U. (2020). Milk as a Complex Multiphase Polydisperse System: Approaches for the Quantitative and Qualitative Analysis. J. Compos. Sci..

[7] Kunes R., Bartos P., Iwasaka G.K., Lang A., Hankovec T., Smutny L., Cerny P., Poborska A., Smetana P., Kriz P. (2021). In-Line Technologies for the Analysis of Important Milk Parameters during the Milking Process: A Review. Agriculture.

[8] Gudkov S.V., Sarimov R.M., Astashev M.E., Pishchalnikov R.Y., Yanykin D.V., Simakin A.V., Shkirin A.V., Serov D.A., Konchekov E.M., Ogly G.-Z.N.G. (2023). Modern Physical Methods and Technologies in Agriculture. Phys. Usp..

[9] Gudkov S.V., Antipov A.V., Astashev M.E., Baybursky V.L., Baimler I.V., Beldova D.A., Bunkin A.F., Burmistrov D.E., Validov S.Z., Vasilyev V.A. (2025). Ecology of Industrial Cities: Non-Standard Scientific and Technological Solutions for Environmental Monitoring, Neutralization, and Subsequent Advanced Processing of Industrial and Municipal Waste (A Review). Russ. J. Gen. Chem..

[10] Liu N., Qi J., An X., Wang Y. (2023). A Review on Information Technologies Applicable to Precision Dairy Farming: Focus on Behavior, Health Monitoring, and the Precise Feeding of Dairy Cows. Agriculture.

[11] Burmistrov D.E., Pavkin D.Y., Khakimov A.R., Ignatenko D.N., Nikitin E.A., Lednev V.N., Lobachevsky Y.P., Gudkov S.V., Zvyagin A.V. (2021). Application of Optical Quality Control Technologies in the Dairy Industry: An Overview. Photonics.

[12] Gastélum-Barrios A., Soto-Zarazúa G.M., Escamilla-García A., Toledano-Ayala M., Macías-Bobadilla G., Jauregui-Vazquez D. (2020). Optical Methods Based on Ultraviolet, Visible, and Near-Infrared Spectra to Estimate Fat and Protein in Raw Milk: A Review. Sensors.

[13] Uusitalo S., Diaz-Olivares J., Sumen J., Hietala E., Adriaens I., Saeys W., Utriainen M., Frondelius L., Pastell M., Aernouts B. (2021). Evaluation of MEMS NIR Spectrometers for On-Farm Analysis of Raw Milk Composition. Foods.

[14] Hass R., Münzberg M., Bressel L., Reich O. (2013). Industrial Applications of Photon Density Wave Spectroscopy for In-Line Particle Sizing [Invited]. Appl. Opt..

[15] Zhu H., Fu H., Yan P., Li X., Zhang L., Wang X., Chai C. (2022). Study on the Release of GMZ Bentonite Colloids by Static Multiple Light Scattering Technique. Colloids Surf. A Physicochem. Eng. Asp..

[16] Shkirin A.V., Ignatenko D.N., Chirikov S.N., Vorobev A.V., Gudkov S.V. (2022). Application of Laser Polarimetric Scatterometry in the Study of Water-Based Multicomponent Bioorganic Systems on the Example of Cow Milk. Phys. Wave Phenom..

[17] Kucheryavskiy S., Melenteva A., Bogomolov A. (2014). Determination of Fat and Total Protein Content in Milk Using Conventional Digital Imaging. Talanta.

[18] Katsumata T., Aizawa H., Komuro S., Ito S., Matsumoto T. (2020). Quantitative Analysis of Fat and Protein Concentrations of Milk Based on Fibre-Optic Evaluation of Back Scattering Intensity. Int. Dairy J..

[19] Wang X., Ke L., Lai S.C., Zhu Q., Sun X.Q., Chua S.J. (2023). Determination of Milk Content by a Laser Light Scattering Technique. J. Mater. Sci. Mater. Electron..

[20] Shkirin A.V., Astashev M.E., Ignatenko D.N., Suyazov N.V., Chirikov S.N., Kirsanov V.V., Pavkin D.Y., Lobachevsky Y.P., Gudkov S.V. (2023). A Monoblock Light-Scattering Milk Fat Percentage and Somatic Cell Count Sensor for Use in Milking Systems. Sensors.

[21] Singh H., Gallier S. (2017). Nature’s Complex Emulsion: The Fat Globules of Milk. Food Hydrocoll..

[22] Shkirin A.V., Ignatenko D.N., Chirikov S.N., Bunkin N.F., Astashev M.E., Gudkov S.V. (2021). Analysis of Fat and Protein Content in Milk Using Laser Polarimetric Scatterometry. Agriculture.

[23] Jain P., Sarma S.E. (2015). Light Scattering and Transmission Measurement Using Digital Imaging for Online Analysis of Constituents in Milk. SPIE Proc..

[24] Runthala A., Mbye M., Ayyash M., Xu Y., Kamal-Eldin A. (2023). Caseins: Versatility of Their Micellar Organization in Relation to the Functional and Nutritional Properties of Milk. Molecules.

[25] Breunig S., Fan Z., Keijzer P., Hettinga K., Bijl E. (2025). Heating Affects Gelation Properties and Casein Micelle Structure in Goat and Cow Milk Differently. Food Struct..

[26] Huppertz T., Gazi I., Luyten H., Nieuwenhuijse H., Alting A., Schokker E. (2017). Hydration of Casein Micelles and Caseinates: Implications for Casein Micelle Structure. Int. Dairy J..

[27] de Kruif C.G., Huppertz T., Urban V.S., Petukhov A.V. (2012). Casein Micelles and Their Internal Structure. Adv. Colloid Interface Sci..

[28] Ménard O., Ahmad S., Rousseau F., Briard-Bion V., Gaucheron F., Lopez C. (2010). Buffalo vs. Cow Milk Fat Globules: Size Distribution, Zeta-Potential, Compositions in Total Fatty Acids and in Polar Lipids from the Milk Fat Globule Membrane. Food Chem..

[29] Shkirin A.V., Suyazov N.V., Chirikov S.N., Chaikov L.L., Shermeneva M.A., Gudkov S.V. (2024). Features of Light Scattering in Turbid Media As Modeled for Two-Component Emulsions. Phys. Wave Phenom..

[30] Roy B., Guha P., Bhattarai R., Nahak P., Karmakar G., Chettri P., Panda A.K. (2016). Influence of Lipid Composition, pH, and Temperature on Physicochemical Properties of Liposomes with Curcumin as Model Drug. J. Oleo Sci..

[31] Beliciu C.M., Moraru C.I. (2009). Effect of Solvent and Temperature on the Size Distribution of Casein Micelles Measured by Dynamic Light Scattering. J. Dairy Sci..

[32] Moroi Y., Nishikido N., Uehara H., Matuura R. (1975). An Interrelationship between Heat of Micelle Formation and Critical Micelle Concentration. J. Colloid Interface Sci..

[33] Verma R., Pyreddy S., Redmond C.E., Qazi F., Khalid A., O’Brien-Simpson N.M., Shukla R., Tomljenovic-Hanic S. (2023). Detection and Identification of Amino Acids and Proteins Using Their Intrinsic Fluorescence in the Visible Light Spectrum. Anal. Chim. Acta.

[34] Yang H., Xiao X., Zhao X., Wu Y. (2017). Intrinsic Fluorescence Spectra of Tryptophan, Tyrosine and Phenyloalanine. SPIE Proc..

[35] Martinho J.M.G., Santos A.M., Fedorov A., Baptista R.P., Taipa M.A., Cabral J.M.S. (2007). Fluorescence of the Single Tryptophan of Cutinase: Temperature and pH Effect on Protein Conformation and Dynamics. Photochem. Photobiol..

[36] Gorokhov V.V., Korvatovsky B.N., Knox P.P., Grishanova N.P., Goryachev S.N., Pashchenko V.Z., Rubin A.B. (2021). Temperature dependence of tryptophan fluorescence lifetime as an indicator of its microenvironment dynamics. Dokl. Biochem. Biophys..

[37] Stevenson K.L., Papadantonakis G.A., LeBreton P.R. (2000). Nanosecond UV Laser Photoionization of Aqueous Tryptophan: Temperature Dependence of Quantum Yield, Mechanism, and Kinetics of Hydrated Electron Decay. J. Photochem. Photobiol. A Chem..

[38] Gooran N., Kopra K. (2024). Fluorescence-Based Protein Stability Monitoring—A Review. Int. J. Mol. Sci..

[39] Paschenko V.Z., Gorokhov V.V., Knox P.P., Korvatovsky B.N., Grishanova N.P., Goryachev S.N., Rubin A.B. (2025). The Spectral and Kinetic Characteristics of Tryptophan Fluorescence in Human and Bovine Serum Albumin at Different Temperatures. Biophysics.

[40] Mills O.E. (1976). Effect of Temperature on Tryptophan Fluorescence of β-Lactoglobulin B. Biochim. Biophys. Acta (BBA)-Protein Struct..

